# Dynamics of *BCR::ABL1* transcript levels and clinical outcomes after switch to second‐line therapy in pediatric chronic myeloid leukemia

**DOI:** 10.1002/hem3.70347

**Published:** 2026-03-24

**Authors:** Stephanie Sembill, Thomas Zerjatke, Micha Winterstein, Tabita Ghete, Elke Schirmer, Zofia Wotschofsky, Meinolf Suttorp, Manuela Krumbholz, Axel Karow, Ingmar Glauche, Markus Metzler

**Affiliations:** ^1^ Pediatric Oncology and Hematology, Department of Pediatrics and Adolescent Medicine University Hospital Erlangen, Friedrich‐Alexander‐Universität Erlangen‐Nürnberg (FAU) Erlangen Germany; ^2^ CCC Erlangen‐EMN: Comprehensive Cancer Center Erlangen‐EMN (CCC ER‐EMN) Erlangen Germany; ^3^ CCC WERA: Comprehensive Cancer Center Alliance WERA (CCC WERA) Erlangen Germany; ^4^ BZKF: Bavarian Cancer Research Center (BZKF) Erlangen Germany; ^5^ Institute for Medical Informatics and Biometry, Carl Gustav Carus Faculty of Medicine TUD Dresden University of Technology Dresden Germany; ^6^ Pediatric Hemato‐Oncology, Carl Gustav Carus Faculty of Medicine TUD Dresden University of Technology Dresden Germany

Since its approval in 2003, imatinib has maintained its status as the primary first‐line tyrosine kinase inhibitor (TKI) for children and adolescents diagnosed with chronic myeloid leukemia (CML).^1^, ^2^ Second‐generation (2G)‐TKIs, including dasatinib and nilotinib, were not approved for pediatric use until 2017 and 2018, respectively, despite their earlier availability for adult patients.^3^ Consequently, imatinib remained the most widely used first‐line agent due to the significantly greater long‐term clinical experience, while 2G‐TKIs have primarily been used as second‐line therapy in cases of resistance or intolerance.^4^

Randomized first‐line adult trials have shown faster and deeper molecular responses with 2G‐TKIs compared with imatinib; corresponding pediatric data from randomized studies are lacking.^5^, ^6^ However, Phase II trials in children with newly diagnosed chronic‐phase CML (CML‐CP) demonstrated similar trends, with 12‐month major molecular response (MMR) rates of approximately 50%–65% under 2G‐TKIs, versus 30%–40% typically reported with imatinib.^7^, ^8^, ^9^, ^10^, ^11^ These findings have encouraged an expanded use of 2G‐TKIs in pediatric patients, not only for resistance or intolerance, but also to achieve deeper molecular remissions (DMR). However, there is a paucity of comprehensive real‐world data on the frequency, rationale, and outcomes of treatment switches in pediatric CML, which engenders uncertainty about which patients benefit most from therapy modifications.

To address this gap, we present herein the analysis of data from the German CML‐paed II trial and registry, focusing on the clinical parameters and molecular outcomes of pediatric patients switching from imatinib to 2G‐TKIs. The CML‐paed II trial was a prospective, investigator‐initiated, multicenter, open‐label Phase III study evaluating upfront imatinib therapy in children and adolescents with CML. The study was approved by institutional ethics committees (EK282 122 006; EK 236_18 B), registered at EUDRACT (2007‐001339‐69) and ClinicalTrials.gov (NCT00445822), and conducted in accordance with the Declaration of Helsinki. Diagnoses and molecular response assessments were performed in certified reference laboratories according to European LeukemiaNet (ELN) criteria.^12^

Patient characteristics were summarized using descriptive statistics and expressed as numbers and percentages or medians for categorical and continuous variables, respectively. Differences in baseline characteristics were assessed using the Mann–Whitney *U* test for continuous data and Fisher's exact test for categorical data. To evaluate molecular response dynamics following the switch, segmented linear regression (“broken‐stick”) models were fitted to individual time courses, with the switch defined as time point 0 and log‐transformed *BCR::ABL1* transcript levels analyzed before and after this point.^13^ Patients with at least two molecular assessments in both time windows were included. Observation periods of ±6 months were used for early switches (≤12 months after TKI initiation) and ±12 months for later switches. Slopes before and after the switch were compared using paired *t*‐tests. Cumulative incidence functions were estimated for the time of achievement of MR3 and MR4, with switch to third‐line therapy treated as a competing risk. Group comparisons were performed using Gray's test. Statistical analyses were performed using *Mathematica* (v14.1, Wolfram Research) and *R* (v4.4.3, R Foundation for Statistical Computing).

From May 2006 to December 2022, 212 pediatric patients were enrolled, including 189 diagnosed with CML‐CP who received imatinib as first‐line treatment.

During follow‐up (median 3.5 years; range, 0–15 years), 70 patients (37%) switched to a 2G‐TKI, predominantly to dasatinib (*n* = 66; 94%) and rarely to nilotinib (*n* = 4; 6%). No progression to the advanced phase or deaths were observed in the switch cohort during the study period. A flowchart illustrating the study population is provided in Figure S1.

The size of the dasatinib group (“Dasa cohort”) allowed for a detailed analysis of this subcohort. The median age at diagnosis was 12 years (range, 3–18 years), and the median time to switch from imatinib was 16 months (range, 3–106 months). In total, 26 patients (39%) switched during the first year of treatment. In line with the year of drug licensing, these patients were diagnosed more recently (median year: 2020; range: 2008–2022) compared to the 40 patients who switched later (median year: 2014; range: 2006–2022). In most patients, therapy was switched due to a non‐favorable molecular response according to ELN criteria, comprising 25 patients (38%) classified as unfavorable and 27 patients (41%) as warning. *BCR::ABL1* kinase domain mutation status was not incorporated into response categorization due to the incomplete availability of mutation analyses.

Eight patients (12%) changed their treatment due to adverse events, including growth retardation (*n* = 2), bone pain (*n* = 2), muscle cramps (*n* = 1), sweating and tremor (*n* = 1), diarrhea (*n* = 1), and rash (*n* = 1). Six patients (9%) switched for other reasons, four of whom aimed to achieve a DMR, while lack of compliance (*n* = 1) and pre‐existing, likely TKI‐unrelated, persistent papilledema (*n* = 1) accounted for the remaining cases. Among the 52 patients with a non‐favorable response, *ABL1* kinase domain mutation analysis was performed using bidirectional Sanger sequencing in 28 patients (54%), revealing resistance‐associated mutations in 6 patients (21%; Figure S2).

Of the 66 patients in the Dasa cohort, 49 met predefined data requirements and were included in the segmented regression analysis (“Follow‐up Dasa cohort”). Characteristics of these patients did not differ from those excluded (Table S1). Most patients (*n* = 40; 82%) switched due to a non‐favorable molecular response, while nine (18%) changed therapy for adverse events or other reasons. Subsequent response analyses focused on the 40 patients with inadequate molecular response. Among these, 18 (45%) were categorized as “unfavorable” and 22 (55%) as “warning” at the time of therapy switch. Baseline demographic and disease characteristics did not differ between patients classified as warning and those classified as unfavorable (Table S2; Figure S3).

Figure 1A illustrates exemplary molecular response trajectories for two patients with unfavorable responses, with the time of switch defined as 0 on the *x*‐axis. Log‐transformed *BCR::ABL1/ABL1* ratios before and after switching are modeled by segmented regression, allowing direct comparison of the slopes before and after the switch. Figure 1B shows regression lines for all patients who switched due to unfavorable response, supplemented by boxplots in Figure 1C addressing the difference in slope initiated by the therapy switch. Segmented regression analysis demonstrated a significant improvement in molecular response kinetics, independent of the timing of the switch. The majority of patients in the unfavorable subgroup (*n* = 15; 83%) benefited from the treatment switch, showing accelerated molecular response dynamics, corresponding to a more pronounced decline in *BCR::ABL1* transcript levels. In contrast, no uniform change in response slope was observed among patients classified as “warning.” Seven patients showed an accelerated response, 10 patients showed a decelerated response, and 5 patients showed virtually no change after switching, reflecting the more heterogeneous nature of this response category (Figure 1D).

**Figure 1:**
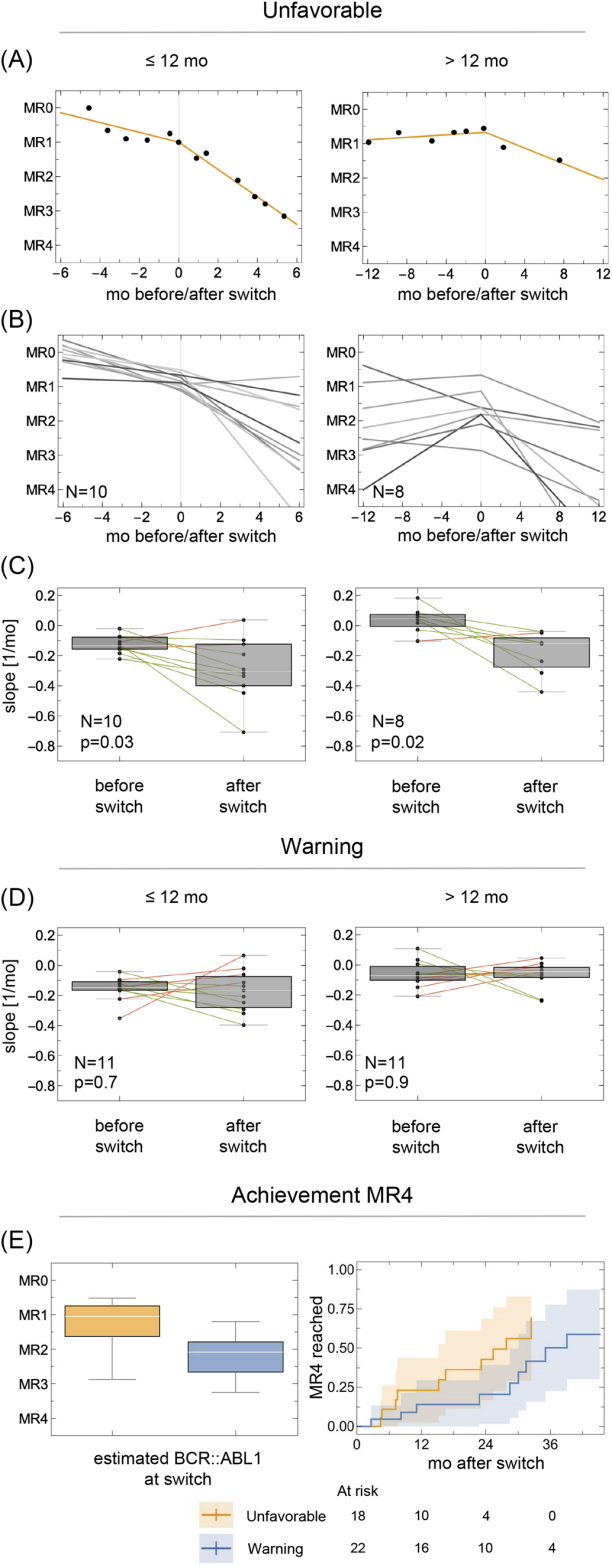
**Dynamics of treatment response and time to achievement of deeper molecular remissions (DMR) after switching to dasatinib. (A)** Exemplary patient molecular response curves illustrating the segmented regression approach to compare slopes before and after the switch. The *x*‐axis shows time in months, with the switch set to “0” (vertical line). The data points on the log(*BCR::ABL1/ABL1*) *y*‐axis (expressed as molecular response [MR] levels) are complemented by two lines derived from a segmented regression model to allow a comparison of the slopes before and after switching. **(B)** Aggregated molecular response trajectories of all patients with an unfavorable response, stratified by timing of the switch (≤12 months vs. >12 months after diagnosis). **(C)** Boxplots showing differences in pre‐ and post‐switch slopes in patients with an unfavorable response; P ≤ 0.05 (paired *t*‐test) considered significant. **(D)** Boxplots comparing slopes before and after switching in patients with a warning response, showing no significant overall change. **(E)** *BCR::ABL1* transcript levels at the time of therapy switch by response category, estimated using segmented regression (left), and cumulative incidence of MR4 after therapy switch stratified by response category (right), with switch to third‐line therapy treated as a competing event. mo, months.

Given the distinct kinetic patterns observed after switching, we next assessed whether these short‐term differences were reflected in long‐term molecular response outcomes. Cumulative rates of achieving MMR (MR3) and DMR (MR4) were analyzed after switching in patients with “warning” or “unfavorable” responses. At the time of switch, *BCR::ABL1* transcript levels were significantly higher in the unfavorable group (P < 0.001; Figure 1E). Despite this, in competing risk analyses, the time to achievement of MMR was comparable between groups (median 10 vs. 8 months; Figure S4). For DMR, a trend toward earlier achievement in the unfavorable group was observed (median 28 vs. 35 months), without reaching statistical significance (Figure 1E). Figures S5–S7 show the molecular response trajectories of all patients who switched treatment for response‐related reasons.

Although limited by the sample size, attributable to the low prevalence of CML in children, this study provides the first real‐world analysis of treatment patterns and outcomes of 2G‐TKIs in pediatric CML. We found that more than one‐third (37%) of children and adolescents initially treated with imatinib switched to a 2G‐TKI, predominantly dasatinib. This switching frequency aligns with adult data, where overall rates range from 25% to 45%, with higher frequencies among patients starting on imatinib.^14^, ^15^, ^16^ In the prospective SIMPLICITY study (NCT01244750), which evaluated first‐line TKI use in adults, 42% of patients switched therapy within 5 years, most commonly due to adverse events (57%) rather than resistance.^17^ In contrast, the rationale for treatment modification in pediatric patients differed markedly: approximately 80% of switches in our cohort occurred due to an inadequate molecular response, whereas intolerance was a minor cause. This distinction likely reflects the lower prevalence of comorbidities in children. While a higher baseline disease burden could contribute to more inadequate responses, our cohort showed an EUTOS long‐term survival (ELTS) score distribution comparable to adult populations with approximately two‐thirds of patients classified as low risk. However, it should be noted that certain pediatric‐specific disease characteristics, such as higher leukocyte counts or spleen size relative to age, are not fully captured by the ELTS score (Table 1).

**Table 1:** Demographic, hematologic, cytogenetic, and treatment characteristics of the 66 patients switched to dasatinib during the course of treatment (Dasa cohort) and the 49 patients who were eligible for the molecular response analysis (Follow‐up Dasa cohort).

	Dasa cohort (*N* = 66)	Follow‐up Dasa cohort (*N* = 49)
*Demographic data*		
Age at dx, years, median [range]	12 [3–18]	12 [5–18]
Sex, f, *N* (%)	26 (39)	20 (40)
Year of dx, median [range]	2016 [2006–2022]	2018 [2006–2022]
*ELTS score*		
High, *N* (%)	7 (11)	4 (8)
Intermediate, *N* (%)	11 (17)	7 (14)
Low, *N* (%)	36 (54)	31 (64)
No data, *N* (%)	12 (18)	7 (14)
*Hematology at dx*		
Leukocytes (10E9/L), median [range]	292.00	324.46
	[13.00–808.00]	[15.32–808.00]
Platelets (10E9/L), median [range]	482.50	457.00
	[165.20–1296.00]	[165.20–1296.00]
Hb (g/dL), median [range]	8.3 [4.9–14.0]	8.3 [5.3–12.6]
*Rearrangement/transcript*		
e13/a2, *N* (%)	17 (26)	9 (18)
e14/a2, *N* (%)	21 (32)	15 (31)
e13/a2 and e14/a2, *N* (%)	8 (12)	7 (14)
No data, *N* (%)	20 (30)	18 (37)
*Cytogenetics*		
*BCR::ABL* only, *N* (%)	46 (70)	34 (69)
Variant translocation, *N* (%)	6 (9)	5 (10)
Additional chromosomal abnormalities, *N* (%)	1 (2)	0 (0)
Complex karyotype, *N* (%)	2 (3)	1 (2)
No data, *N* (%)	11 (16)	9 (19)
*Clinical data/follow‐up*		
Switch 2nd line ≤ 12 months (%)	26 (39)	23 (47)
Time from dx to switch (months), median [range]	16 [3–106]	12 [3–97]
Reason for switch “unfavorable,” *N* (%)	25 (38)	18 (37)
Reason for switch “warning,” *N* (%)	27 (41)	22 (45)
Reason for switch side effects, *N* (%)	8 (12)	4 (8)
Other reasons for switch, *N* (%)	6 (9)	5 (10)
Follow‐up from dx (years), median [range]	4 [0–15]	3 [1–8]
Follow‐up from switch (months), median [range]	29 [0–180]	28 [4–72]

Abbreviations: dx, diagnosis; ELTS score, EUTOS long‐term survival score; Hb, hemoglobin.

The increasing frequency of early switches in recent years reflects improved access to pediatric 2G‐TKIs and growing clinical confidence in their use. Dasatinib was the preferred second‐line agent due to its once‐daily dosing and the advantage that it did not have to be taken on an empty stomach.

Our findings indicate that patients who fail to respond to first‐line imatinib therapy can benefit substantially from therapy switching, reflected by an immediate improvement in response kinetics after the switch and a trend toward earlier attainment of DMR. For pediatric patients, achieving DMR is especially important because the desired treatment goals extend beyond an optimal response. Due to the significance of long‐term, treatment‐related adverse effects, such as growth disturbances, it is crucial to optimize treatment strategies for this population. Since achieving DMR is a prerequisite for approaching treatment‐free remission, it underscores the importance of optimizing treatment strategies early in the disease course.

In contrast, patients categorized as “warning” showed heterogeneous outcomes. The absence of a significant change in the slope after switching, along with the comparatively later achievement of MR4, suggests that some of these patients may generally have a slower molecular response, independent of the TKI used, representing so‐called “late responders.” These results underscore the necessity of individualized approaches and adapted therapeutic strategies for this subgroup.

## AUTHOR CONTRIBUTIONS

**Stephanie Sembill**: Writing—original draft; conceptualization; methodology; validation; visualization; funding acquisition; data curation; writing—review and editing. **Thomas Zerjatke**: Writing—original draft; methodology; validation; visualization; writing—review and editing; formal analysis. **Micha Winterstein**: Writing—review and editing; methodology; data curation. **Tabita Ghete**: Writing—review and editing; methodology; data curation. **Elke Schirmer**: Data curation; writing—review and editing; methodology. **Zofia Wotschofsky**: Writing—review and editing; methodology. **Meinolf Suttorp**: Writing—review and editing; methodology. **Manuela Krumbholz**: Writing—review and editing; methodology. **Axel Karow**: Writing—review and editing; methodology; conceptualization. **Ingmar Glauche**: Writing—original draft; writing and editing; conceptualization; methodology; formal analysis; visualization; validation. **Markus Metzler**: Writing—original draft; conceptualization; visualization; methodology; formal analysis; writing—review and editing.

## CONFLICT OF INTEREST STATEMENT

The authors declare no conflicts of interest.

## ETHICS STATEMENT

CML‐PAED‐II was an investigator‐initiated, academic‐supported, multicenter, open‐label, single‐arm Phase III clinical trial that recruited from March 2004 to December 2015 and registered at EUDRACT‐2007‐001339‐69 and ClinicalTrials.gov (NCT00445822). The protocol was approved by the institutional ethics committee (EK282 122 006). The subsequent registry is authorized by the institutional ethics committee (EK 236_18 B).

## FUNDING

The study was supported by a research grant from the ELAN fund (ELAN 23‐04‐19‐1‐Sembill to S.S.), Interdisciplinary Center for Clinical Research (IZKF) at the University Hospital of the University of Erlangen‐Nürnberg. S.S. was further supported by the IZKF through the clinician scientist program. The continuous financial support in data collection by “Sonnenstrahl e.V. Dresden ‐ Förderkreis für krebskranke Kinder und Jugendliche” (Dresden, Germany), “Schornsteinfeger helfen krebskranken Kindern e.V.” (Dörfles‐Esbach, Germany), and the “Madeleine‐Schickedanz‐Kinderkrebsstiftung” (Fürth, Germany) is highly acknowledged. Open Access funding enabled and organized by Projekt DEAL.

## Supporting information

Supporting Information.

## Data Availability

The data that support the findings of this study are available from the corresponding author upon reasonable request.
