# Supplementary material for: Dynamics of BCR::ABL1 transcript levels and clinical outcomes after switch to second‐line therapy in pediatric chronic myeloid leukemia
Source: Hemasphere. 2026 Mar 24;10(3):e70347. doi: 10.1002/hem3.70347 (PMC13045481; doi:10.1002/hem3.70347)

**Supplemental Material**

referring to the article

**Dynamics of *BCR::ABL1* Transcript Monitoring and Clinical Outcomes after Switch to Second-Line Therapy in Pediatric Chronic Myeloid Leukemia**

**Content:**

Supplemental Table 1**.** Comparison of baseline characteristics between patients included in the segmented regression analysis (“Follow-Up Dasa cohort”) and those excluded from the analysis.

Supplemental Table 2: The distribution of ELTS risk categories within the two subgroups “unfavorable” and “warning”.

Supplemental Figure 1. Study flowchart.

Supplemental Figure 2. Distribution of the 7 *ABL1* kinase domain mutations.

Supplemental Figure 3. Comparison of baseline and clinical characteristics between the “warning” and “failure” subgroups.

Supplemental Figure 4. Time to achievement of major- molecular- response.

Supplemental Figure 5. Individual molecular response trajectories of patients who switched therapy due to potential to achieve deeper molecular remission.

Supplemental Figure 6. Individual molecular response trajectories of all patients who switched due to unfavorable response.

Supplemental Figure 7. Individual molecular response trajectories of all patients who switched due to warning response.

**Supplemental Table 1.**

Comparison of baseline characteristics between patients included in the segmented regression analysis (“Follow-Up Dasa cohort”) and those excluded from the analysis. For comparison Mann-Whitney U tests (metric data) and Fisher’s exact tests (categorial data) was used.

|  | Follow-Up Dasa cohort  (N=49) | Excluded patients  (N=17) | p-value |
| --- | --- | --- | --- |
| **Demographic data** |  |  |  |
| Age at dx, years, median [range] | 12 [5-18] | 12 [3-16] | 0.648 |
| Sex, f, N (%) | 20 (41) | 6 (35) | 0.778 |
| **Hematology at dx** |  |  |  |
| Leukocytes (10E9/L), median [range] (N=63) | 324.46  [15.32-808.00] | 228.00  [13.00-635.00] | 0.129 |
| Platelets (10E9/L), median [range] (N=59) | 457.00  [165.20-1296.00] | 520.50  [172.00- 832.00] | 0.742 |
| Hb (g/dl), median [range] (N=61) | 8.3  [5.3-12.6] | 8.5  [4.9-14.0] | 0.909 |
| **Rearrangement/Transcript (N=46)** |  |  |  |
| e13/a2, N (%) | 9 (29) | 8 (53) | 0.238 |
| e14/a2, N (%) | 15 (48) | 6 (40) |  |
| e13/a2 and e14/a2, N (%) | 7 (23) | 1 (7) |  |
| **Cytogenetics**  **(N=55)** |  |  |  |
| *BCR::ABL* only, N (%) | 34 (85) | 12 (80) | 0.256 |
| Variant translocation, N (%) | 5 (13) | 1 (7) |  |
| Complex karyotype, N (%) | 1 (3) | 1 (7) |  |
| ACA, N (%) | 0 (0) | 1 (7) |  |

ACA=additional chromosomal aberration, dx=diagnosis, Hb= hemoglobin

**Supplemental Table 2.**

The distribution of ELTS risk categories within the two subgroups “unfavorable” and “warning”.

|  | Unfavorable  (N=25) | Warning  (N=27) |
| --- | --- | --- |
| **ELTS Score** |  |  |
| High, N (%) | 3 (12) | 3 (11) |
| Intermediate, N (%) | 4 (16) | 7 (25) |
| Low, N (%) | 12 (48) | 12 (44) |
| No data, N (%) | 6 (24) | 5 (20) |

**Supplemental Figure 1**

Study flowchart. **
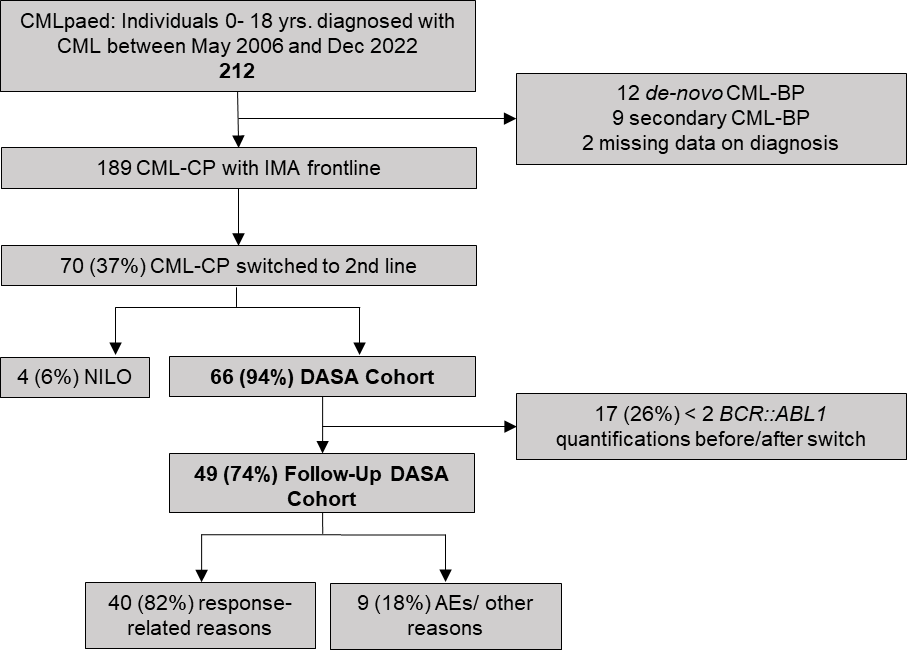
**

From May 2006 to December 2022, 212 pediatric patients were diagnosed with CML in Germany. Of those patients, 189 received imatinib as their first-line therapy. During treatment, 70 patients (37%) switched to second-line therapy, and 66 of them (94%) received dasatinib (DASA cohort). Molecular response analysis included 49 patients (follow-up DASA cohort). 40 (82%) transitioned to dasatinib due to response-related reasons ("warning" or "unfavorable"), while nine (18%) switched due to adverse events or other reasons.

AE = adverse event; BP = blast phase; CML = chronic myeloid leukemia; CP = chronic phase; DASA = dasatinib; IMA = imatinib; NILO = nilotinib.

**Supplemental Figure 2**

Distribution of the 7 *ABL1* kinase domain mutations.

**
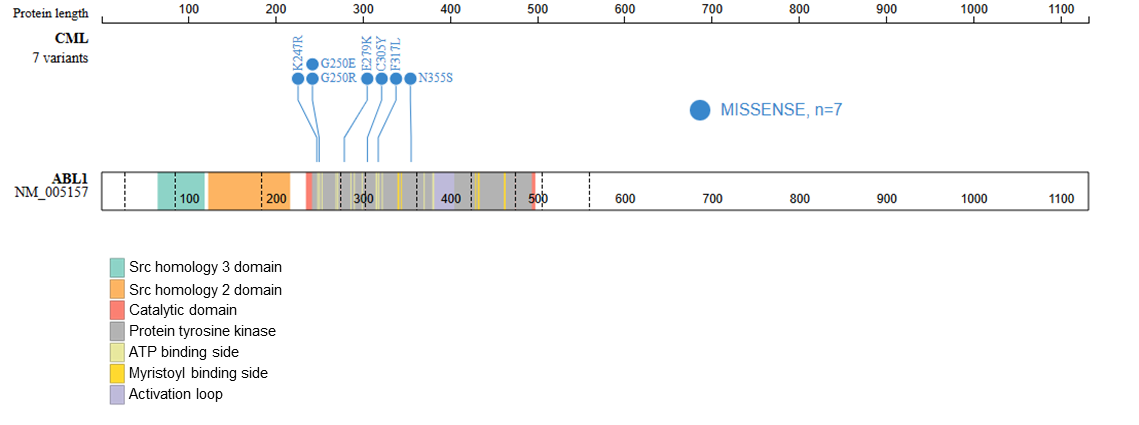
**

*ABL1* kinase domain mutations detected in 6 out of 28 patients in whom mutation analysis was performed.

**Supplemental Figure 3**

Comparison of baseline and clinical characteristics between the “warning” and “failure” subgroups.


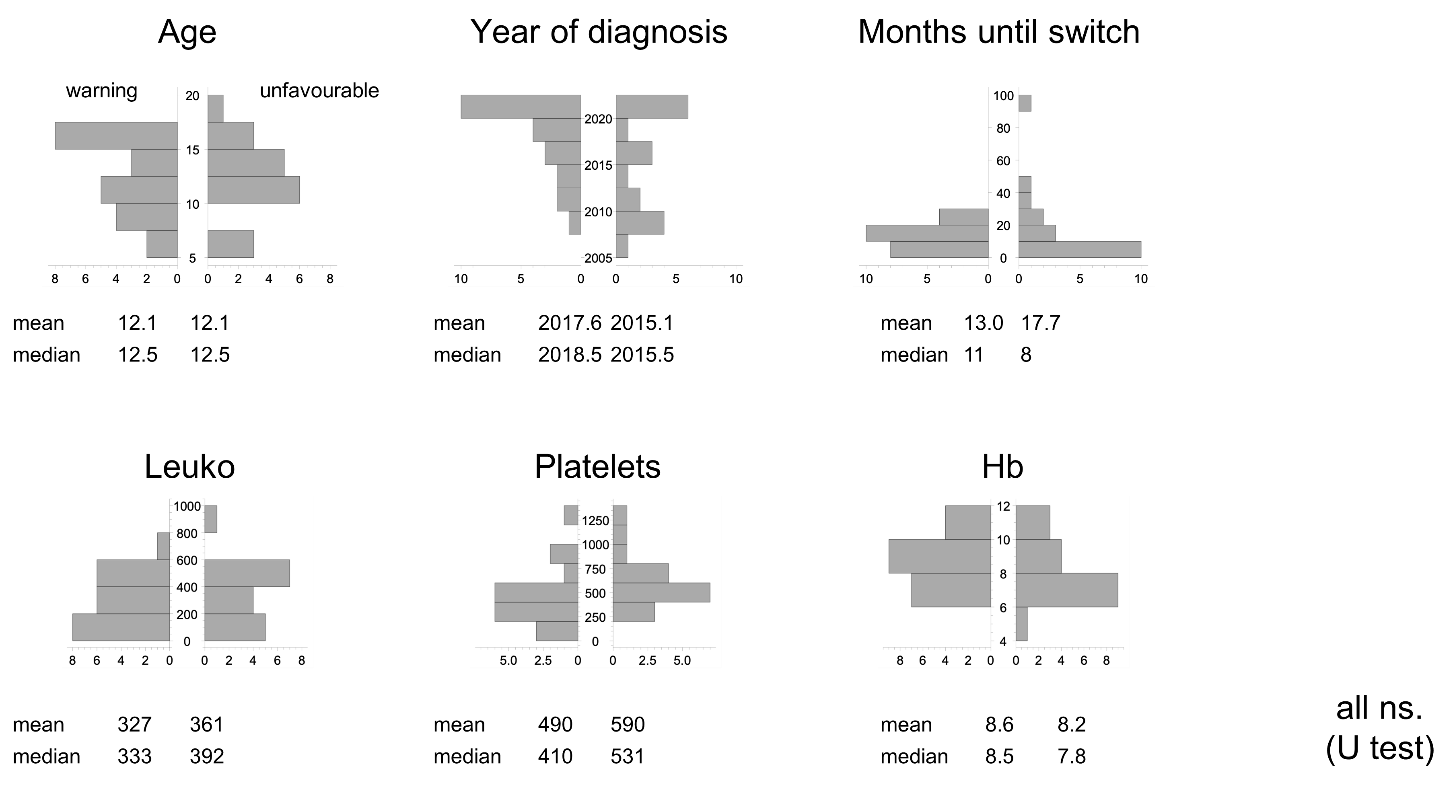


Comparison of the two subgroups “warning” and “failure” with regard to potential factors influencing the molecular outcome. The statistical analysis was performed using the U test.

**Supplemental Figure 4**

Time to achievement of major molecular response.


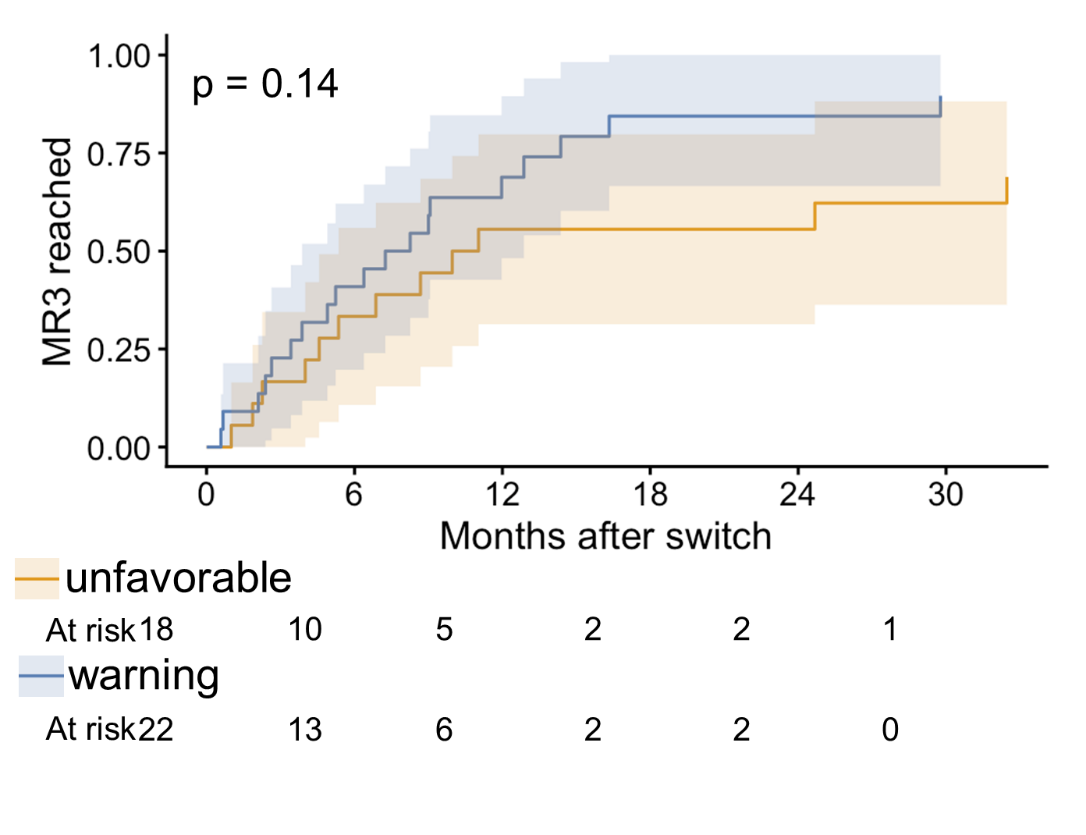


Cumulative incidence of MR3 after therapy switch stratified by response category, with switch to third-line therapy treated as a competing event

mo = months

**Supplemental Figure 5**

Individual molecular response trajectories of patients who switched therapy due to potential to achieve deeper molecular remission.


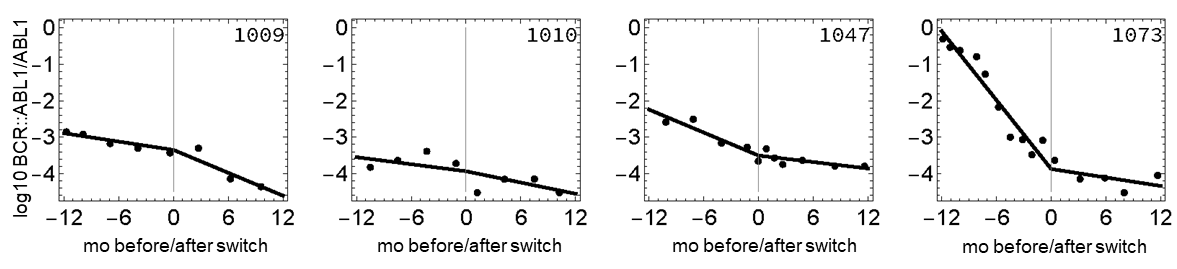


Comparisons of the slopes before and after therapy switch showed no significant differences.

**Supplemental Figure 6**

Individual molecular response trajectories of all patients who switched due to unfavorable response


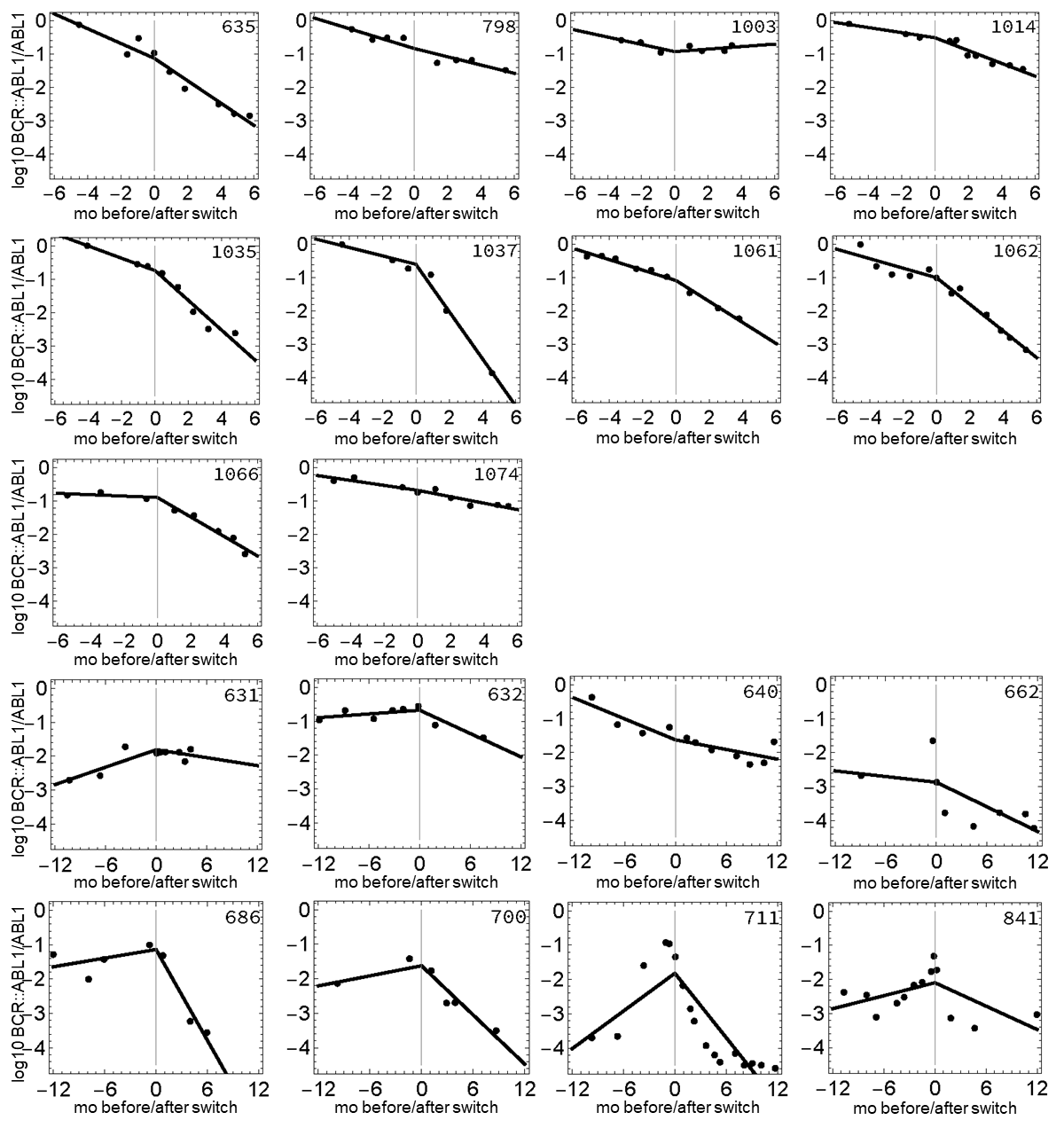


**Supplemental Figure 7**

Individual molecular response trajectories of all patients who switched due to warning response


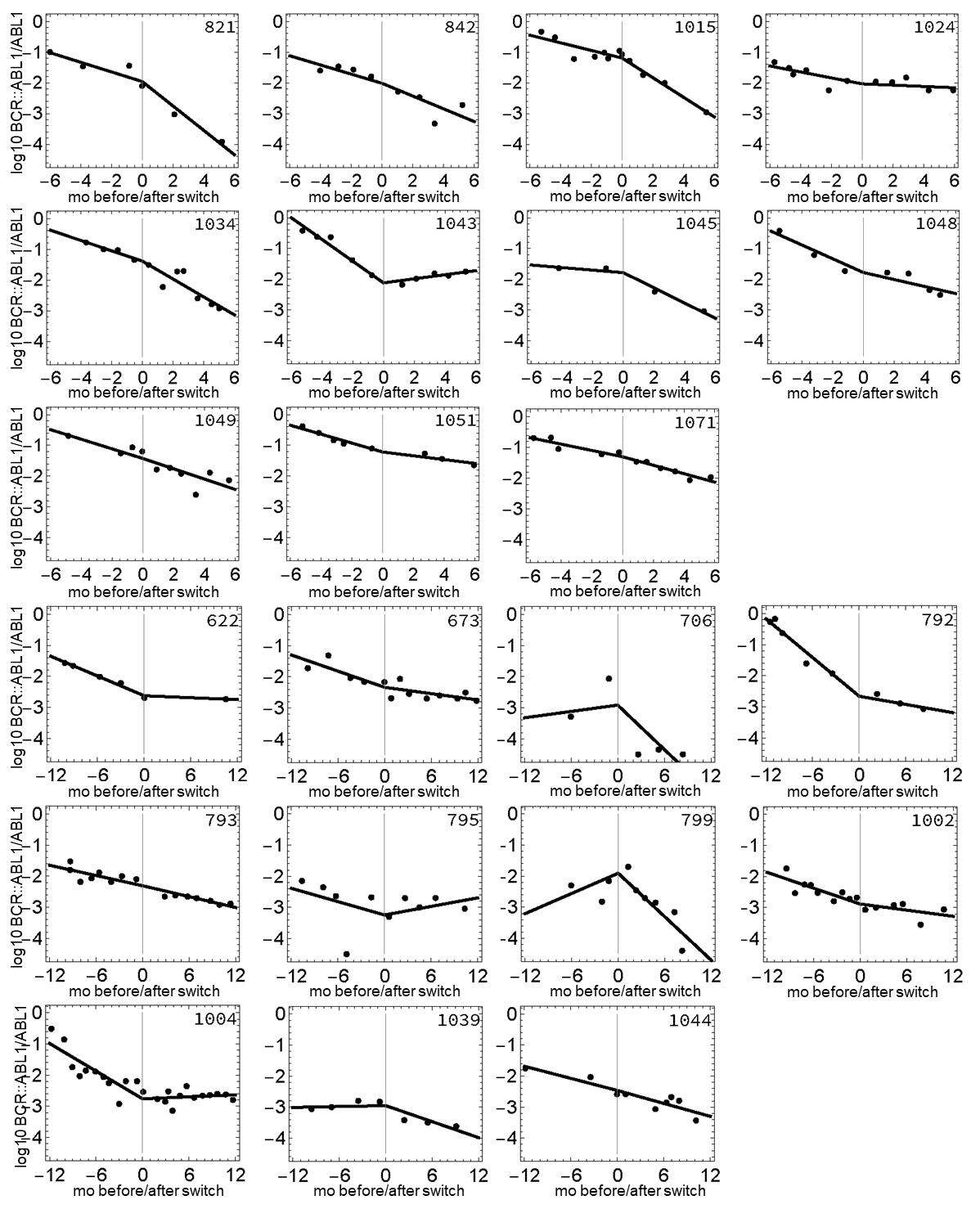

Supplement: Supplementary file 1 — Supporting Information. [file HEM3-10-e70347-s001.docx]
